# Case Report: Management of multiple brown tumors after kidney transplantation

**DOI:** 10.3389/fendo.2025.1645715

**Published:** 2025-09-16

**Authors:** Qingyi Sun, Yafei Bai, Ruman Chen, Hong Li

**Affiliations:** Blood Purification Center, Hainan General Hospital/Hainan Medical University Affiliated Hainan Hospital, Haikou, Hainan, China

**Keywords:** brown tumors, kidney transplantation, secondary hyperparathyroidism, parathyroid hormone, case report

## Abstract

Brown tumor is usually caused by primary or secondary hyperparathyroidism but is exceptionally rare after kidney transplantation. Their rarity and atypical pathological features make diagnosis and treatment particularly challenging. We present a case of a 38-year-old woman with multiple brown tumors secondary to persistent hyperparathyroidism following kidney transplantation. The patient was initially admitted to the orthopedic department for left shoulder dislocation and lytic lesions in the left humerus and was diagnosed with a giant cell tumor of bone. Further investigations revealed elevated parathyroid hormone (PTH) levels and multiple lytic bone lesions throughout the skeleton. Based on these findings, the patient was ultimately diagnosed with multiple brown tumors. As a result, the patient underwent total parathyroidectomy and autotransplantation of parathyroid tissue. Follow-up evaluations showed decreased PTH levels and alkaline phosphatase levels, with improvement in skeletal changes. This case report shares the experience and lessons in managing hyperparathyroidism, both before and after kidney transplantation, emphasizing the importance of clinicians’ awareness of the disease and multidisciplinary collaborative management.

## 1 Background

Brown tumor is rare complications of both primary and secondary hyperparathyroidism, occurring in only 2–5% of affected individualsgt (1). Kidney transplantation(KT) is the optimal treatment for end-stage renal disease.It was previously believed that secondary hyperparathyroidism would resolve after kidney transplantation as the transplanted kidney’s function is restored. However, about 25% of recipients still show symptoms of secondary hyperparathyroidism (SHPT) 1 year after transplantation (2). Brown tumor, as a late manifestation of hyperparathyroidism, usually indicate a prolonged duration or more severe progression of the disease. These skeletal changes can lead to fractures and subsequent disability, imposing a heavy burden on patients. The diagnosis of brown tumor relies on clinical findings, laboratory tests, imaging studies, and pathological examination. However, these results are often nonspecific, complicating diagnosis and treatment. This article presents a case of diagnostic and therapeutic challenges of brown tumor following KT, aiming to enhance clinicians’ awareness of this condition and reduce the risk of misdiagnosis.

## 2 Case presentation

**Figure 1:**
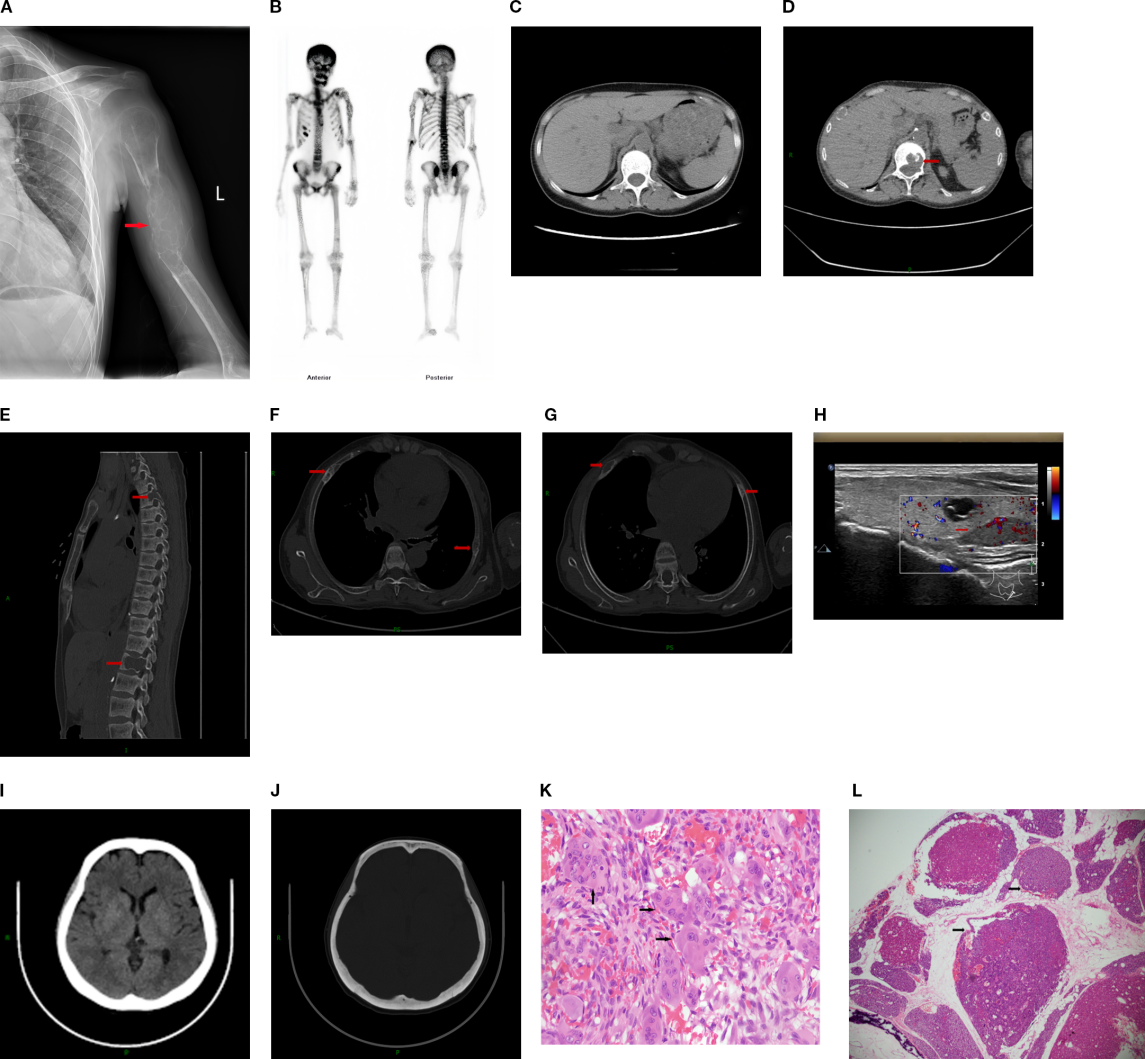
**(A)** Left humeral X–ray Examination revealing Osteolytic lesion with a “soap bubble” appearance in the left humerus, accompanied by a pathological fracture. **(B)** Whole–Body Bone Scan: Abnormal bone metabolism in the left humerus, skull, and right 5th and 6th costal cartilages. **(C)** The mediastinal window of the patient's pulmonary CT scan reveals no bone destruction in the first lumbar vertebra. **(D, E)** Thoracic CT and lumbar spine CT demonstrate bone destruction in the first lumbar vertebra,Spine CT also shows bone destruction in the third thoracic vertebra. **(F, G)** Rib CT showing multiple bone cortex discontinuities in multiple ribs. **(H)** Parathyroid Ultrasound with parathyroid hyperplasia. **(I, J)** Cranial CT demonstrates no intracranial abnormalities and shows intact calvarial bone. **(K)** Histopathology of left humeral (haematoxylin–eosin (HE) staining, magnification ×40. **(L)** Histopathology of parathyroid gland (haematoxylin–eosin (HE) staining, magnification ×2.

**Figure 2:**
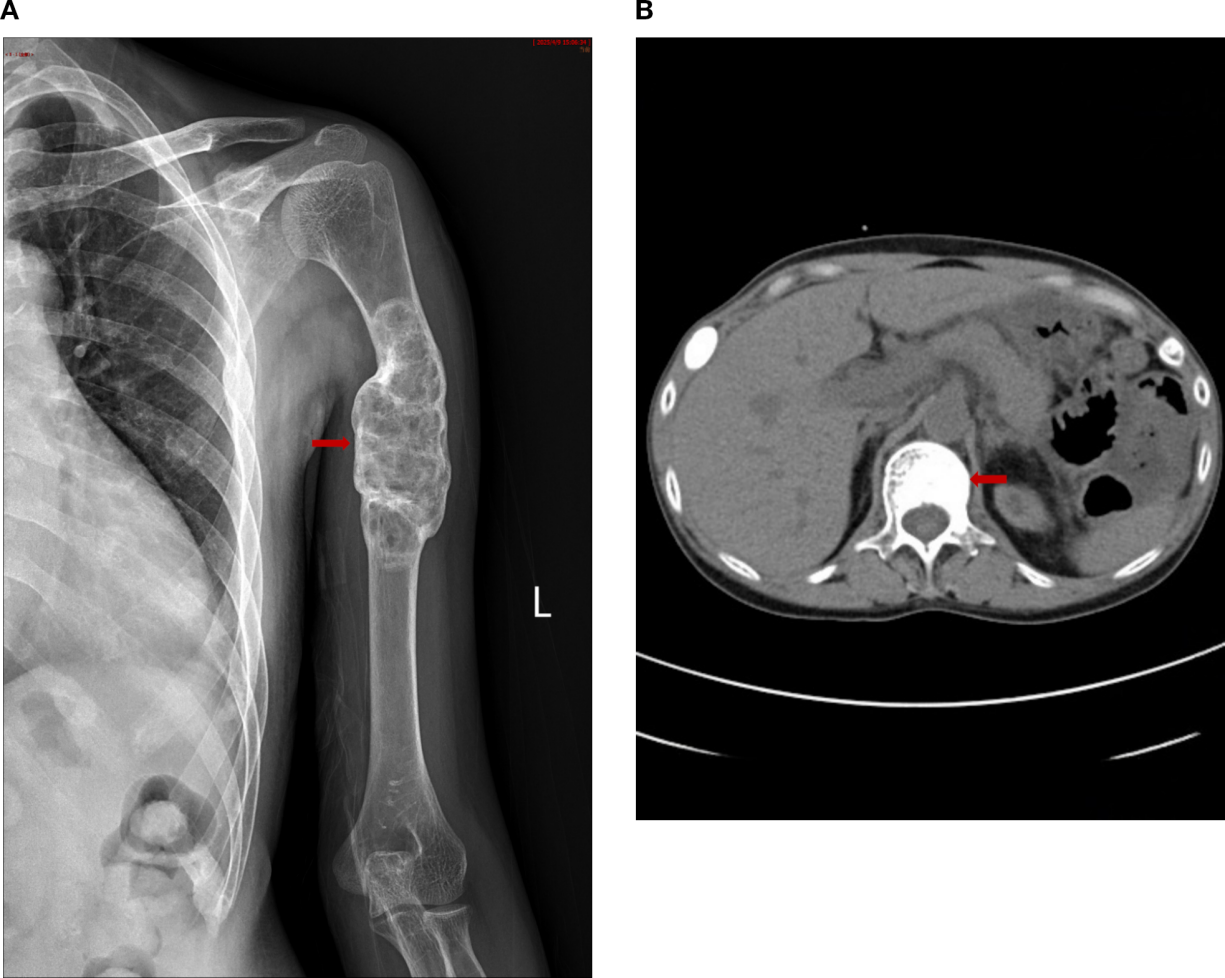
**(A)**: Left humeral X–ray Examination revealing: Increased density of the lesion, thickened cortical bone, no definite signs of fracture, and improvement of the previous left shoulder dislocation. **(B)** The lumbar vertebral defect has shown improvement compared to the previous condition.

Female, 38 years old. She presented with persistent left upper arm pain for 2 months, limiting mobility, and was admitted to the orthopedics department in July 2024. She had undergone KT in March 2022 after 4 years of hemodialysis. During hemodialysis, her intact parathyroid hormone (iPTH) levels ranged from 1253 to 1595 pg/ml. She had been treated with paricalcitol and cinacalcet but discontinued these medications due to hypercalcemia and severe gastrointestinal reactions. The patient had concurrent hyperphosphatemia and was non-compliant with medical advice due to anticipation of spontaneous resolution of SHPT post-kidney transplantation. However, iPTH levels remained at approximately 2000 pg/ml post-transplant. The patient was subsequently maintained on long-term oral calcitriol at a dose of 0.25 µg daily. Physical examination revealed that her blood pressure was 134/84 mmHg. The patient had an anemic appearance. The left forearm was sling, limiting movement of the left shoulder joint. There was significant local tenderness, with no other positive physical signs. Laboratory tests revealed that the hemoglobin level was 69 g/L and that the creatinine level was 237μmol/L. Left humeral X-ray Examination showed an osteolytic lesion in the middle–upper segment of the left humerus, suggesting neoplastic changes, along with a pathological fracture and dislocation of the left shoulder joint (**Figure 1A**). Whole-Body Bone Scan: Abnormal bone metabolism in the left humerus, skull, and right 5th and 6th costal cartilages (**Figure 1B**). However, PTH levels were not checked during this hospitalization. The patient was discharged after a left humeral puncture biopsy. Pathology revealed osteoclasts distributed in a sheet-like pattern, with many fibroblasts observed in the interstitium. Immunohistochemistry results: H3.3G34W (-). The diagnosis was a giant cell tumor of the bone (**Figure 1K**). The patient was readmitted to the orthopedics department with a diagnosis of giant cell tumor of bone. During the preoperative examination, compared with the pulmonary CT scan performed before renal transplantation 2 years ago (**Figure 1C**), the current CT scan of the lungs, ribs, and spine reveals new osteolytic changes in the first lumbar vertebra (**Figures 1D, E**) and the the ribs, suggestive of multiple brown tumors (**Figures 1F, G**). Endocrinology consultation advised completing the assessment of PTH and other laboratory parameters, along with parathyroid ultrasound. Subsequent laboratory tests revealed the following results: iPTH, 1955.6 pg/ml,25-hydroxy vitamin D, 18.7 ng/ml,calcium, 2.27 mmol/L,phosphorus, 0.93 mmol/L,and alkaline phosphatase, 225.1 U/L. A parathyroid ultrasound revealed parathyroid hyperplasia (**Figure 1H**). The cranial CT revealed no intracranial abnormalities or evidence of cranial bone destruction(**Figures 1I, J**). After multidisciplinary consultation, the patient was diagnosed with transplant renal dysfunction, allograft kidney transplantation status,multiple brown tumors secondary to hyperparathyroidism. The patient was subsequently transferred from Orthopedics to our department for continued management. Given the patient’s prior gastrointestinal reactions to oral cinacalcet and their reluctance to regularly visit the hospital for paricalcitol injections, and in line with the patient’s preferences, total parathyroidectomy(RTx) with autotransplantation was performed by an experienced head and neck surgeon after excluding surgical contraindications. The surgery involved complete removal of all parathyroid tissue from the patient. To prevent permanent postoperative hypoparathyroidism and intractable hypocalcemia, a piece of parathyroid tissue without nodular hyperplasia (measuring approximately 1 mm × 1 mm × 1 mm) was selected and transplanted into the patient’s right forearm. Postoperative pathology of the resected parathyroid tissue revealed nodular hyperplasia. (**Figure 1L**). To prevent postoperative hypocalcemia, the patient received a postoperative infusion of 10% calcium gluconate at 10 ml/h via an infusion pump. Additionally, the patient was prescribed oral calcium carbonate 1.5 g (three times daily) and calcitriol 1 µg (four times daily). Blood calcium levels were checked every 4 hours, and the infusion rate was adjusted accordingly. The infusion was stopped once blood calcium levels stabilized, and the dosage of oral medications was then adjusted based on blood calcium levels. Postoperative monitoring revealed iPTH on the non-transplanted extremity between 19.5 – 251.4pg/mL, calcium levels between 2.08 - 2.74 mmol/L, phosphorus levels between 0.65 - 0.76 mmol/L, and alkaline phosphatase levels at 188.1 -191.7U/L. The patient was discharged on the fourth postoperative day.The patient failed to return to our hospital for the scheduled systematic postoperative RTx follow-up and did not take medications on time. Around 8 months postoperatively, the patient developed diarrhea and self-discontinued calcium carbonate and calcitriol. Blood tests at another hospital revealed a drop in calcium levels to 1.33 mmol/L and an increase in iPTH to 1181 pg/mL. However, the limb from which the final blood draw was taken could not be confirmed. It is speculated that blood may have been mistakenly drawn from the transplanted limb. Concurrently, serum creatinine rose to 601. Renal biopsy of the transplanted kidney revealed polyomavirus -associated nephropathy (PVAN), acute severe T cell-mediated rejection (ASTB3), and Baff class 3; glomerular endothelial injury was noted, with antibody-mediated rejection (ABMR) not excluded, prompting a recommendation for digital subtraction angiography (DSA). Subsequently, re-examination at our hospital revealed PTH levels of 157 pg/mL on the non-transplanted side and 1511 pg/mL on the transplanted side. Additionally, alkaline phosphatase was 97.5 U/L, serum calcium was 1.3mmol/L, serum phosphate was 1.76 mmol/Land 25-Hydroxyvitamin D was 18.2ng/ml (**Table 1**). Ultrasound of the neck and the mass in the right upper limb showed no evidence of hyperplastic parathyroid tissue. Left humeral X-ray examination showed increased density of the lesion and thickened cortical bone compared with the previous X-ray. There were no definite signs of fracture, and the previous left shoulder dislocation had improved (**Figure 2A**).The lumbar vertebral defect has shown improvement compared to the previous condition (**Figure 2B**). The patient had no limitation in the movement of the left joint and no clinical manifestations such as limb pain and difficulty in walking. For treatment, temporary administration of intravenous calcium gluconate was given. Vitamin D was administered at 5000 IU once daily, calcium carbonate at 1.5 g three times daily, and calcitriol at 1 µg three times daily. Three days later, the rechecked serum calcium level had risen to 2.01 mmol/L.As for the orthopedic aspect, it is recommended to continue the current conservative treatment and follow up regularly.

**Table 1:** Preoperative and postoperative biochemical markers of bone metabolism.

	preop	Day1	Day2	Day3	About 2 months	About 8 months	Rang
iPTH(Pg/ml)	1955.6	251(NTE)	19.5(NTE)	65.6(NTE)	153(NT) 1798(TE)	157(NTE) 1511(TE)	15–68.3
ALP(U/L)	225.1	192	188.1	‐	93	97.5	35–100
Calcium(mmol/l)	2.35	2.74	2.36	2.08	2.0	1.3	2.11–2.52
Phosphorus(mmol/L)	0.7	0.76	0.65	0.65	1.02	1.76	0.85–1.51
Serum Creatinine(umol/L)	237	–	–	–	409	630	41–73
25–Hydroxyvitamin D(ng/ml)	18.7	14.6	–	–	–	18.2	>30.0

iPTH, intact Parathyroid Hormone; ALP, Alkaline Phosphatase; preop, Preoperative; NTE, Non–transplanted extremity; TE, Transplanted extremity.

## 3 Discussion

KT is an important treatment option for patients with chronic renal failure. The current literature indicates that kidney transplant recipients typically have extended life expectancies, enhanced quality of life, and decreased societal costs compared with those on the waiting list (3–5). Abnormal bone metabolism often persists after kidney transplantation. Contributing factors include pre–transplant renal osteodystrophy, glucocorticoids, calcineurin inhibitors (CNIs), persistent hyperparathyroidism, and deficiencies of calcium and vitamin D (6, 7). The incidence of persistent hyperparathyroidism in kidney transplant recipients can reach up to 50% (8). Persistent hyperparathyroidism in kidney transplant recipients can be classified into two types: SHPT and tertiary hyperparathyroidism.

(THPT).A prospective study by Whitney Sutton et al. found that the incidence of SHPT was 61.7%, while that of THPT was 21.5% in kidney transplant recipients (9). There are no diagnostic criteria for tertiary hyperparathyroidism. Some scholars believe that its clinical manifestations include uncontrollable hypercalcemia, which suggests monoclonal or oligoclonal proliferation of parathyroid cells (10). However, some scholars suggest that it can also be defined as persistent PTH elevation 6 months post–transplantation, with or without hypercalcemia (11). Hypercalcemia typically results from parathyroid hormone (PTH)–enhanced renal tubular calcium reabsorption, calcitriol–mediated gastrointestinal calcium absorption, and potential PTH–induced bone calcium release. In this case, the patient had normal corrected calcium levels without hypercalcemia, likely due to post–transplant renal dysfunction and vitamin D deficiency. Persistent hyperparathyroidism in kidney transplant recipients is attributed to the long–term persistence of parathyroid enlargement, the prolonged regression of parathyroid hyperplasia, residual renal dysfunction post–transplantation, and certain genetic factors. Among these, polymorphisms in the vitamin D receptor (VDR) gene are believed to play a significant role. Several genetic variants of the VDR have been identified, including FokI (rs2228570), TaqI (rs731236), BsmI(rs1544410), ApaI (rs7975232) (12). The ApaI❝C”allele, TaqI❝tt”genotype, BsmI❝BB” genotype, and FokI variant have all been linked to elevated PTH levels (13–15). The BsmI “bb” genotype is associated with elevated PTH levels, decreased calcitriol levels, and accelerated progression of SHPT in pre–dialysis CKD patients and kidney transplant recipients (16). Increased parathyroid hormone levels production results in hypercalcemia (17). leading to bone demineralization, microfractures, hemorrhage, hemosiderin deposition (18) and excessive vascular proliferation. These changes give the lesions their characteristic brown staining, justifying the nomenclature “brown tumor(BT)”.However, The pathogenesis of brown tumor remains incompletely understood. Turek D et al. conducted targeted sequencing in 16 patients with BT and identified pathogenic KRAS mutations in nearly two–thirds of the cases (19). Vitamin D deficiency may exacerbate the clinical manifestations of brown tumors (20). Considering the role of vitamin D receptor (VDR) polymorphisms in hyperparathyroidism, it is plausible that VDR polymorphisms also contribute to the development of brown tumors. However, research in this area is currently lacking, and this may represent an important direction for future investigations into the pathogenesis of brown tumor. There is a gender difference in the incidence of BT, with studies showing a higher prevalence in females, at a male–to–female ratio of 3:18 (21). BT can occur in any bone, presenting as either solitary or multiple lesions. Recent case reports have highlighted the rare occurrence of brown tumor deposits in the orbit (22). They present diverse symptoms on the basis of the size and location of the bone lesions (23). Clinically, they may be asymptomatic or cause pain and fractures. Laboratory tests revealed elevated serum PTH levels, decreased serum phosphate, normal or elevated alkaline phosphatase, elevated serum calcium, and vitamin D deficiency. Imaging findings of brown tumors can present as diffuse osteopenia, osteoporosis, bone deformities, and circumscribed osteolytic lesions (24). A recent study has shown that 99mTc–MIBI imaging is valuable in differentiating thyroid cancer metastases from brown tumors (25). This examination is recommended to be performed before surgery to aid in accurate diagnosis and guide appropriate treatment strategies. Histopathologic ally, the lesion is characterized by clusters of multinucleated giant cells, fibrous tissue proliferation, and hemosiderin deposits (26). These features can be mistaken for giant cell–rich lesions such as giant cell tumor of bone. Histone H3.3 is encoded by two genes, H3F3A located on chromosome 1 and H3F3B located on chromosome 17, and it plays a crucial role in the pathogenesis and progression of many tumors. Up to 90% of giant cell tumor of bone patients harbor H3F3A G34W mutations (27), and this patient’s genetic test was negative. Therefore, the diagnosis of brown tumors requires the integration of clinical symptoms, laboratory tests, imaging evaluations and pathology. Brown tumor, also known as fibrous osteitis cystica (28), arise from increased PTH secretion, causing an imbalance in osteoclast activity (29). Their treatment focuses on managing PTH levels. Given the importance of vitamin D and VDR polymorphisms in persistent post–renal–transplant hyperparathyroidism, and the high prevalence of vitamin D deficiency after KT, vitamin D supplementation is particularly important following renal transplantation. Supplementation with ergocalciferol (vitamin D2), cholecalciferol (vitamin D3), active vitamin D (calcitriol), or vitamin D analogs (paricalcitol) can be selected based on the recipient’s vitamin D levels and specific clinical condition. Vitamin D treatment can reduce the incidence of persistent post–transplant hyperparathyroidism from 39% to 25% at 1 year (30). In a double–blind, placebo–controlled, randomized trial, 4000 IU/d of cholecalciferol significantly increased vitamin D levels and decreased PTH levels at 12 months post–transplantation compared with placebo (31). Vitamin D receptor activators (VDRAs), such as alfacalcidol, calcitriol, and paricalcitol, can reduce PTH levels and improve bone mineral density (BMD) after transplantation (32). Currently, there is no consensus on the recommended vitamin D supplementation protocols in the field of kidney transplantation. Bonani et al. reported a prospective study demonstrating that biannual denosumab treatment can increase bone mineral density in kidney transplant recipients (33). Additionally, bisphosphonates may help prevent fractures after KT (34). However, there is still a lack of relevant research on drug treatment for brown tumor. In the treatment of SHPT, RTx(subtotal or total) was shown to be more effective compared with those who underwent medical management (35). The surgical treatment for brown tumors is determined by the size and location of the lesion, as well as any associated functional impairment. In a retrospective analysis of 26 cases of brown tumor treatment, two patients with brown tumors in the humerus region had pathological fractures managed solely with a Velpeau bandage. No surgical intervention was required in this area during the 36.1–month follow–up period after RTx. Conversely, one patient with a brown tumor in the femoral neck region experienced a pathological fracture 1 month after RTx and required total hip arthroplasty (36). For spinal lesions causing symptoms due to nerve compression, surgical intervention to relieve the compression is required before RTx (37, 38). After initial RTx, some patients may develop persistent or recurrent hyperparathyroidism. Persistent disease is defined as PTH levels failing to decrease to a specified threshold postoperatively, while recurrent disease is characterized by an initial decrease in serum PTH followed by a subsequent rise, typically occurring 6 months after surgery (11). The etiology of this phenomenon remains unclear. The possible causes include: 1) physiological changes of PTH: Postoperative hypocalcemia activates calcium receptors in parathyroid cells, prompting increased PTH secretion as an adaptive compensatory response.2) hungry bone syndrome (HBS): Physiologic or low doses of PTH promote bone formation. After RTx, the sharp drop in serum PTH levels enhances bone formation. Under the action of osteoblasts, calcium and phosphorus from the blood are rapidly deposited into the bone, leading to rapid and persistent hypocalcemia, HBS (39). 3) vitamin D deficiency and VDR polymorphisms: Research indicates that patients with elevated postoperative PTH levels have lower vitamin D levels compared to those with normal PTH levels (40). Calcitriol suppresses PTH release via the VDR, leading to direct gene suppression (41). In a study of 121 hemodialysis patients undergoing parathyroidectomy, the BB genotype of BsmI was found to delay the need for surgery, suggesting a protective effect against hyperparathyroidism (42). Additionally, VDR knockout mouse studies demonstrate a direct cellular effect of VDR on osteoblast formation during bone regeneration (43). Research also links VDR gene polymorphisms to an increased risk of post–transplant bone disease (44). In a 5–year follow–up study of 234 kidney transplant recipients, the Cdx2 TT genotype significantly increased the risk of low bone mineral density, while the BsmI CT/TT genotype was linked to a higher risk of avascular necrosis (44). 4) the presence of one or more residual glands. Unfortunately, in this case, PTH levels on the non–transplanted side did not normalize postoperatively. Potential causes include vitamin D deficiency, hypocalcemia, and the possible presence of undetected ectopic parathyroid tissue. Regarding the management of the brown tumor in this case, our 8–month follow–up results demonstrate significant improvement in bone destruction of the left humerus and first lumbar vertebra following total parathyroidectomy and auto transplantation. Thus, we currently deem orthopedic intervention unnecessary for this patient. Nevertheless, long–term follow–up remains essential.

## 4 Conclusion

Brown tumor is extremely rare after kidney transplantation and often lead to misdiagnosis or missed diagnosis due to their atypical symptoms. It can cause fractures and subsequent disabilities, significantly affecting patient outcomes. This underscores the diagnostic challenges faced by clinicians. Future efforts should focus on developing precise diagnostic and treatment protocols to improve the prognosis for patients with brown tumor.

## Data Availability

The datasets presented in this study can be found in online repositories. The names of the repository/repositories and accession number(s) can be found in the article/supplementary material.
